# Glycation and Oxidative Stress Increase Autoantibodies in the Elderly

**DOI:** 10.3390/molecules25163675

**Published:** 2020-08-12

**Authors:** Mohd W.A. Khan, Ahmed Al Otaibi, Subuhi Sherwani, Wahid A. Khan, Eida M. Alshammari, Salma A. Al-Zahrani, Mohd Saleem, Shahper N. Khan, Sultan Alouffi

**Affiliations:** 1 Department of Chemistry, College of Sciences, University of Ha’il, Ha’il 2440, Saudi Arabia; ahmed.alotaibi@uoh.edu.sa (A.A.O.); eida.alshammari@uoh.edu.sa (E.M.A.); s.alzahrane@uoh.edu.sa (S.A.A.-Z.); 2 Molecular Diagnostic and Personalised Therapeutics Unit, University of Ha’il, Ha’il 2440, Saudi Arabia; s.alouffi@uoh.edu.sa; 3 Department of Biology, College of Sciences, University of Ha’il, Ha’il 2440, Saudi Arabia; s.sherwani@uoh.edu.sa; 4 Department of Clinical Biochemistry, College of Medicine, King Khalid University, Abha 62529, Saudi Arabia; wkhan@kku.edu.sa; 5 Department of Pathology, Sub-Division of Medical Microbiology, College of Medicine, University of Ha’il, Ha’il 2440, Saudi Arabia; m.saleem@uoh.edu.sa; 6 Interdisciplinary Nanotechnology Centre, Aligarh Muslim University, Aligarh 202002, India; snkhan_co@myamu.ac.in; 7 Department of Clinical Laboratory Sciences, College of Applied Medical Sciences, University of Ha’il, Ha’il 2440, Saudi Arabia

**Keywords:** aging, glycation, AGEs, oxidative stress, natural products, autoantibodies, elderly

## Abstract

Aging causes gradual changes in free radicals, antioxidants, and immune-imbalance in the elderly. This study aims to understand links among aging, gluco-oxidative stress, and autoantibodies in asymptomatic individuals. In vitro glycation of human serum albumin (Gly-HSA) induces appreciable biochemical changes. Significant inhibition of advanced glycation end products (AGEs) formation was achieved using garlic extract (53.75%) and epigallocatechin-3-gallate from green tea (72.5%). Increased amounts of serum carbonyl content (2.42 ± 0.5) and pentosidine (0.0321 ± 0.0029) were detected in IV-S (S represent smokers) vs. IV group individuals. Direct binding ELISA results exhibited significantly high autoantibodies against Gly-HSA in group IV-S (0.55 ± 0.054; *p* < 0.001) and III-S (0.40 ± 0.044; *p* < 0.01) individuals as compared to the age matched subjects who were non-smokers (group IV and III). Moreover, high average percent inhibition (51.3 ± 4.1%) was obtained against Gly-HSA in IV-S group individuals. Apparent association constant was found to be high for serum immunoglobulin-G (IgG) from group IV-S (1.18 × 10^−6^ M) vs. serum IgG from IV group (3.32 × 10^−7^ M). Aging induced gluco-oxidative stress and AGEs formation may generate neo-epitopes on blood-proteins, contributing to production of autoantibodies in the elderly, especially smokers. Use of anti-glycation natural products may reduce age-related pathophysiological changes.

## 1. Introduction

Life expectancy has increased throughout the world with increase in medical facilities and research outputs [1]. According to Ferrucci et al., one in four Europeans is projected to be aged 65 years or older by 2030 [2]. According to demographic calculations, between 2000 and 2050, the population of 60 years and older is expected to grow from 605 million to 2 billion people; this increase is subjected to developing countries [3]. There are many age-related changes in older people, such as compromised kidney function, increased risk for cardiovascular diseases, with high cholesterol and connective tissue damage. Clinical parameters are also altered with age [4]; higher levels of plasma glucose are found in the older age groups (>69 years) [5], and glycated hemoglobin (HbA1C) increases with age [6]. Oxidative stress plays a crucial role in the development of age related diseases, and imbalance of several defensive mechanisms that respond to the reactive oxygen species (ROS) inducing cellular damage [7,8]. 

The non-enzymatic formation of advanced glycation end products (AGEs) is observed during normal aging, and occurs endogenously inside—as well as outside—of cells. Glycation reaction contributes to the process of aging. The Maillard reaction products accumulate in long-lived tissue proteins (such as collagens, cartilage, and crystallins) with age, even without any diseases [9,10,11], and are inevitable components of the aging process in all eukaryotic organisms, including humans [12]. The age-related accumulation of AGEs was also shown in pericardial fluid [13]. Reactions of reducing sugars, such as glucose with protein amino groups, are thought to contribute to secondary complications in diabetes, such as nephropathy and vascular disease [14], and lead to similar symptoms in aging [15]. 

Protein bound dicarbonyl compounds are well-known oxidative stress markers formed by the dehydration and rearrangement of Amadori compounds or by spontaneous decomposition of intermediates [9]. Hence, oxidative stress may be involved in the formation of AGEs and vice versa. These AGEs, and their precursors, are the source of free radical generation, providing a mechanism by which AGEs may accelerate oxidative damage to proteins during aging. There is a direct relation of the production of ROS causing oxidative damage to the macromolecules over the lifetime of subjects, which are important in determining the life span of an individual [16]. There is evidence that AGEs have antigenic properties that may induce autoimmune responses [17]. An earlier study has shown the presence of protein AGEs and deposition of their immune complexes in several human tissues, including kidneys in diabetic nephropathic patients [18]. There are also evidences of high prevalence of non-organ specific autoantibodies in the elderly, such as antibodies against rheumatoid factor (14%), antinuclear antibodies (31%), and anti-cardiolipin (51%) detected in healthy individuals over 80 years old [19]. 

It is difficult to reduce the toxicities of AGEs by simply changing lifestyle. Hence, using preventive medicine seems to be the best approach to preventing damage caused by glycation. Synthetic medicinal compounds usually have greater side effects, as compared to natural products. Therefore, use of natural products as potential inhibitors of AGEs may be recommended. Many medical herbs exhibit similar levels of anti-glycation properties [20], or sometimes even higher [21] than aminoguanidine, which is a standard inhibitor of glycation [22]. Natural products and their derivatives are important dietary sources that are efficient in anti-oxidant activities and have potential to prevent chronic diseases.

An in vitro glycated form of human serum albumin (HSA) was used as an antigen in this study, as albumin is the most abundant protein present in serum, and has the potential to be affected most, due to any physiological changes linked with aging. Presence of autoantibodies against Gly-HSA (Gly-HSA-Abs) were detected and correlated with age and other gluco-oxidative parameters. 

## 2. Results

### 2.1. Glycation of HSA 

Biochemical studies have been done to measure the extent of glycation of HSA. To detect the early glycation products of Maillard reaction, ketoamines were approximated. The ketoamine moieties generated in the reaction sample of HSA were estimated colorimetrically by using nitroblue tetrazolium (NBT) method (Table 1). The amount of ketoamines was found to be significantly higher (*p* < 0.01) in Gly-HSA (6.4 ± 0.3 mol/mol of HSA) as compared to native HSA (N-HSA) (0.25 ± 0.1 mol/mol of HSA). 

Protein bound carbonyl content comprise the intermediary products of glycation reaction and these were estimated by DNPH reaction, for both non-glycated and glycated HSA samples. Significant amounts (*p* < 0.001) of carbonyl compounds were generated on glucose modification of HSA (3.11 ± 0.4 mol/mol of HSA) as compared to N-HSA (0.1 ± 0.02 nmol/mg protein) (Table 1). 

To further ascertain the complete glycation of HSA, we estimated AGE pentosidine in the reaction samples. A well-known AGE, pentosidine, was estimated by fluorescence spectroscopy using the excitation wavelength at 275 nm, which is considered optimum for pentosidine. Gly-HSA exhibited significantly high (*p* < 0.001) pentosidine-specific fluorescence (247.5 ± 8.7 AU). However, a negligible amount was detected in non-glycated HSA (5.2 ± 0.7) (Table 1). Similarly, remarkable differences in pentosidine formation were obtained for Gly-HSA (0.0863 ± 0.012 µg/mL) and N-HSA (0.0092 ± 0.0004 µg/mL) respectively, using ELISA method (data not shown).

### 2.2. Natural Product and Their Derivatives Inhibit AGEs 

**Figure 1:**
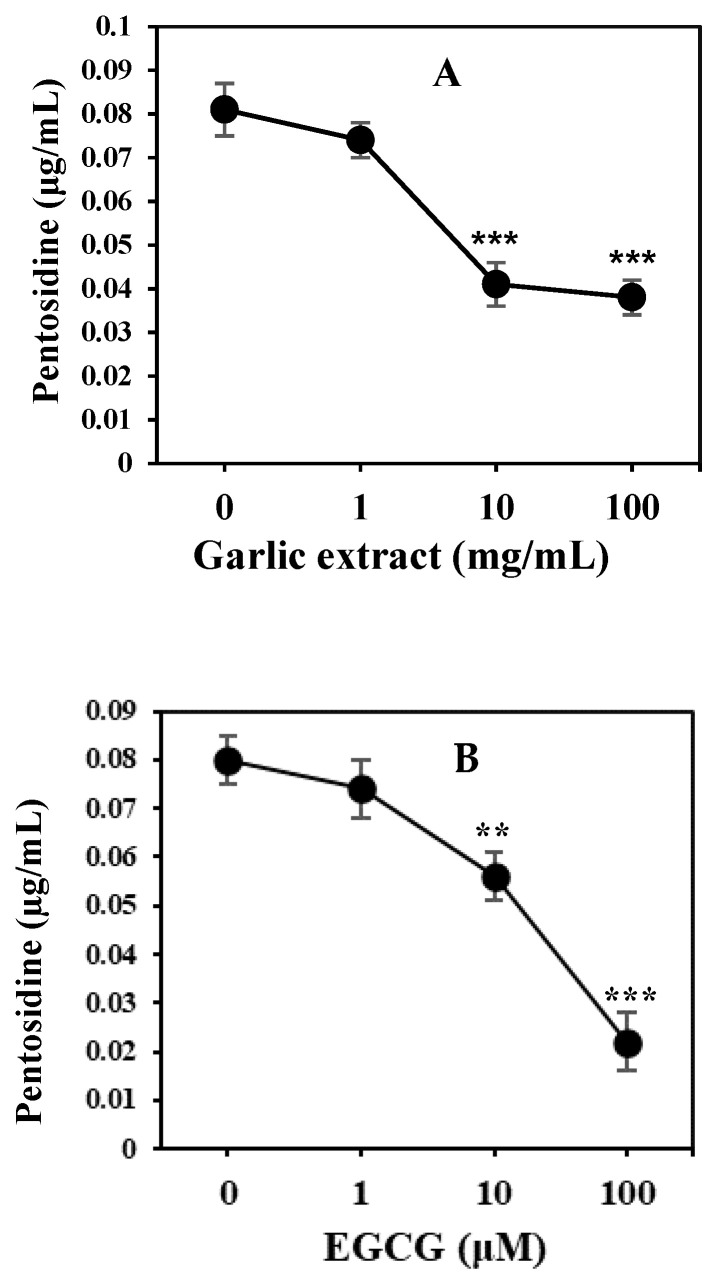
HSA samples (20 µM) were incubated with 50 mM D-glucose in absence and presence of inhibitors garlic extract and epigallocatechin-3-gallate (EGCG) for 10 weeks. Inhibitory effect of garlic extract (1–100 mg/mL) (**A**) and EGCG (1–100 µM) (**B**) on the formation of advanced glycation end products (AGEs) were assayed. Results are presented as mean ± SD (*n* = 3). At each time interval, statistical significances were calculated against glycated samples without inhibitors. *t* test was adopted for the comparison between the two groups and significance is defined as ** *p*  <   0.01, *** *p*  <  0.001. Garlic extract at 10 and 100 mg/mL showed *p* < 0.001 when compared with 0 mg/mL. EGCG at 10 and 100 µM showed *p* < 0.001 and *p* < 0.01 respectively, when compared with 0 µM EGCG.

It is necessary to investigate AGE inhibitors to offer a potential therapeutic approach for the prevention of disorders or pathological complications induced by glycation products. Natural product extracts from garlic and derivative epigallocatechin-3-gallate (EGCG) from green tea have been evaluated as inhibitors against the in vitro formation of AGEs. Garlic extract exhibited maximum inhibition of 53.75% of glycation at 10 ug/mL concentration (Figure 1A). Moreover, EGCG showed maximum inhibition of 72.5% of glycation at 100 µM (Figure 1B). Garlic extract and EGCG exhibited significant inhibition in the formation of AGEs. 

### 2.3. Estimation of Oxidative Stress in Subjects

Protein bound carbonyl content are formed during oxidative stress conditions and are considered a marker of overall protein oxidation. Carbonyl content levels were quantified in all the groups based on age differences and smoking habits (Table 2). Increased levels of carbonyl content (nmol/mg protein) were detected in individuals who were more than 80 years of age and were smokers (group IV-S) (2.42 ± 0.3), followed by 61–80 years and smokers (group III-S) (1.64 ± 0.3), more than 80 years age and non-smokers (group IV) (1.21 ± 0.3), 61–80 years and non-smokers (group III) (0.92 ± 0.22), 41–60 years and smokers (group II-S) (1.2 ± 0.2), 21–40 years and smokers (group I-S) (0.93 ± 0.14), 21–40 years and non-smokers (group II) (0.84 ± 0.14), and 21–40 years and non-smokers (group I) (0.78 ± 0.15). From the results, subject groups who were smokers exhibited higher amount of carbonyl compounds as compared to the non-smokers of same-aged matched groups. There is a gradual increase in the difference of carbonyl contents between groups of aged matched smoker and non-smoker subjects with increased age. An increase in carbonyl content was observed between group II and group II-S. This difference was further significantly increased (*p* < 0.01) with age, as observed in higher age group individuals; group III and III-S. Maximum difference (*p* < 0.001) of carbonyl compounds was observed in subjects who were more than 80 years of age and were smokers (IV-S group) as compared to the age matched nonsmoker individuals (IV group) (Table 2). The difference in serum carbonyl content between the age-matched groups, with or without smokers, increased with increasing age. This showed that less amounts of carbonyl contents were generated in non-smoking groups with increased age as compared to the subjects with a history of smoking. Moreover, there were no remarkable difference in serum carbonyl content between males and females within the group. 

### 2.4. Serum Pentosidine Estimation

Pentosidine concentrations were estimated in serum samples for all the groups (Table 2). Significantly increased levels were estimated in subjects from IV-S (0.0321 ± 0.0031 µg/mL) and III-S (0.0285 ± 0.0031 µg/mL) as compared to their age matched subjects from IV (0.0271 ± 0.0027 µg/mL) and III (0.0265 ± 0.0022 µg/mL) respectively, who were non-smokers. Subjects from II-S (0.0269 ± 0.0029 µg/mL) also showed an increased pentosidine concentration as compared to the subjects from group II, III-S and I. Moreover, there were no significant differences between male and female results within the group.

### 2.5. Proinflammatory Cytokines in Serum

Proinflammatory cytokines interleukin-1β (IL-1β) and interleukin-6 (IL-6) were estimated in serum samples of all the subjects from different groups. Significant increase in the levels of both IL-1β (1.62 ± 0.21 pg/mL; *p* < 0.05) and IL-6 (7.2 ± 0.65 pg/mL; *p* < 0.001) were detected in individuals who were smokers and more than 80 years of age (IV-S) as compared to the same age group subjects who were non-smokers (1.20 ± 0.19 pg/mL) and (5.2 ± 0.54 pg/mL), respectively (Table 3). Moreover, serum levels of IL-6 exhibited significant increase (6.4 ± 0.69 pg/mL; *p* < 0.05) in individuals who were more than 60 years of age and were smokers (III-S group) as compared to their age matched group (III) (Table 3). 

### 2.6. Detection of Circulating Gly-HSA-Abs by Direct ELISA 

**Figure 2:**
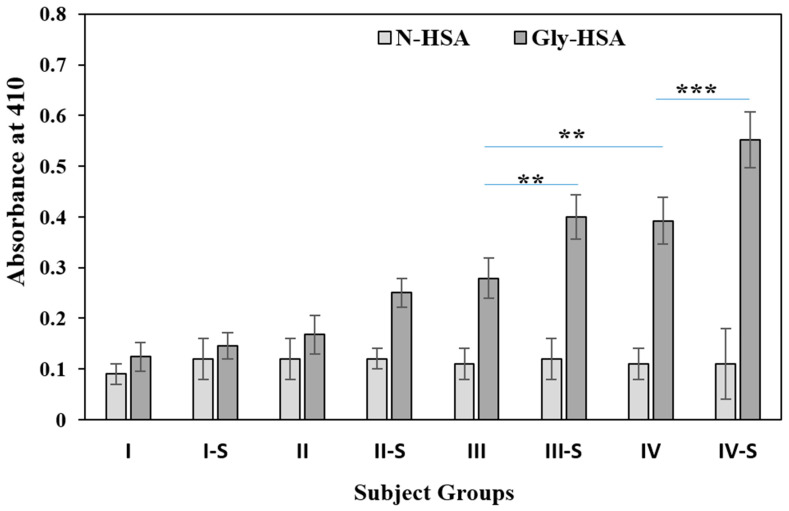
Anti-Gly-HSA-Abs were detected by direct binding ELISA form serum samples (1:100 diluted) of all the subjects in different groups. Groups were divided based on age and smoking habits; group I (21–40), I-S (21–40), II (41–60), II-S (41–60), III (61–80), III-S (61–80), IV (>80) and IV-S (>80). ‘S’ in the group represent subjects who are smokers. Data presented as mean ± SD. *t* test was adopted for the comparison between the two groups and significance is defined as ** *p*  <  0.01, *** *p*  <  0.001. The comparison between group IV-S and IV showed *p* < 0.001. The comparison between III-S and III showed *p* < 0.01. The comparison between IV and III showed *p* < 0.01.

Circulatory autoantibodies from serum samples of all the subjects were screened for non-glycated and glycated HSA proteins. N-HSA and Gly-HSA were used as antigens for the direct binding ELISA assays for all subjects present in this study (Figure 2). Results from direct binding ELISA, showed significantly high binding (*p* < 0.001) of serum autoantibodies in subjects from group IV-S (OD; 0.55 ± 0.054) who were smokers and more than 80 years of age as compared to the non-smoker age matched subjects of group IV (0.39 ± 0.046) (Figure 2). Similarly, significant binding (*p* < 0.05) was observed in group III-S (0.4 ± 0.044) subjects, as compared to group III (0.28 ± 0.040) (Figure 2). However, no significant autoantibody binding differences were observed between groups II-S (0.25 ± 0.029) and II (0.17 ± 0.038) subjects as well as in group I-S (0.14 ± 0.026) vs I (0.12 ± 0.028) subjects. 

Recognition of autoantibody to Gly-HSA antigen in non-smoker individuals from groups IV and III also showed significant differences (*p* < 0.05).

**Figure 3:**
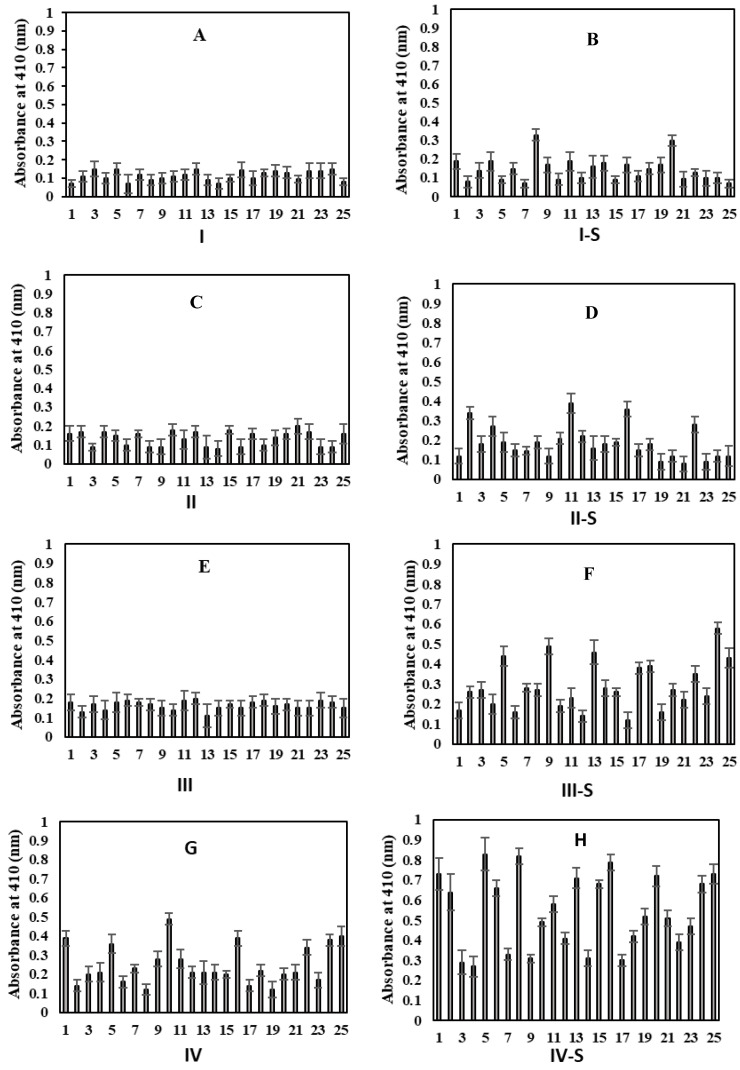
Direct binding ELISA for serum samples (1:100 diluted) for the detection of serum autoantibodies against Gly-HSA from individuals from various groups. Figures A–H represent; groups (**A**): I (21–40), (**B**): I-S (21–40), (**C**): II (41–60), (**D**): II-S (41–60), (**E**): III (61–80), (**F**): III-S (61–80), (**G**): IV (>80), and (**H**): IV-S (>80), respectively. ‘S’ represent subjects who are smokers. Each sample was run in triplicate and data is presented as mean ± SD.

Subjects from all groups showed very low or negligible binding to N-HSA (Figure 2). For a clearer understanding of serum autoantibody binding patterns to the Gly-HSA, direct binding ELISA results for each serum sample from every group is presented in Figure 3.

The correlation analysis between Gly-HSA-Abs and fasting blood glucose (FBG), serum levels of pentosidine, carbonyl content and IL-6 showed strong correlation in different age groups (II, II-S, III, III-S, IV, and IV-S) (Table 4). Pentosidine, carbonyl content, and IL-6 also showed correlation with Gly-HSA-Abs in group I and I-S, however FBG did not show correlation with Gly-HSA-Abs for groups I and I-S. This analysis showed that the data for FBG, pentosidine, carbonyl content, and IL-6 are highly significant and consistent for age groups II, II-S, III, III-S, IV, and IV-S. Thus, Gly-HSA-Abs might exert influence in gluco-oxidation and pro-inflammatory cytokines or vice versa. Hence, concomitant effect of all these factors might induce pathophysiological changes in elderly. 

### 2.7. Inhibition ELISA of Serum Autoantibodies Against Gly-HSA

The binding specificity of the circulating autoantibodies with N-HSA and Gly-HSA was further ascertained by inhibition ELISA using N-HSA and Gly-HSA as inhibitors as given in Table 3. As shown in direct binding ELISA results, Inhibition ELISA of subjects from group IV-S using Gly-HSA as an inhibitor, showed significantly (*p* < 0.001) high maximum percent inhibition (51.3 ± 4.1%) as compared to the age matched subjects from group IV (29.3 ± 4.3%). Significant differences (*p* < 0.05) in the result of inhibition ELISA was observed between subjects of group III-S (33.1 ± 4.0%) and group III (21.1 ± 3.3%). When percent inhibitions were compared between subjects from groups II-S vs. II and subjects from groups I-S vs I, the results were non-significant. Inhibition with N-HSA did not show any appreciable recognition in subjects from all groups (Table 3). 

### 2.8. Quantification of Apparent Association Constant

**Figure 4:**
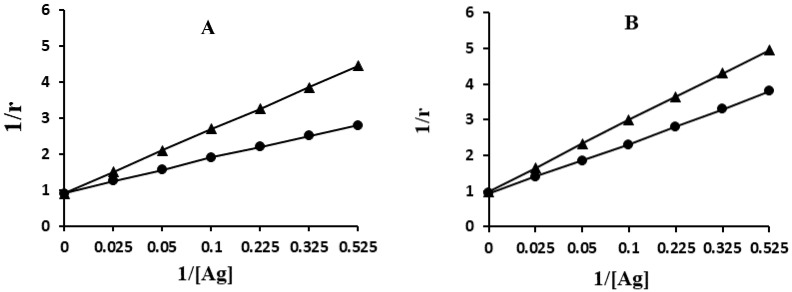
Determination of antigen-antibody binding affinity and apparent association constant by Langmuir plot. Antigen used in this assay were Gly-HSA. Immune complexes were prepared by incubating 100 µg of IgG from IV-S (-●-) and IV (-▲-) subjects (**A**) and IgG from III-S (-●-) and III (-▲-) subjects (**B**) with varying amount of antigen (0–100 mg) in an assay volume of 100 µL for 2 h at room temperature and overnight at 4 °C.

The antigen-antibody interactions of smoker and non-smoker subjects were evaluated by quantitative precipitation titration (Figure 4). In this procedure, serum samples that were showing high direct binding results were selected and age matched.

Serum IgG from (serum no. 5) an individual who was aged more than 80 years and was a smoker (IV-S group), showed 29 µg of Gly-HSA bound to 73 µg of IgG. However, age matched individual (serum no. 10) from non-smoker group (IV group) exhibited 36 µg of Gly-HSA bound to 65 µg of IgG (Figure 4A). Similar pattern of result was observed from an individual (serum no.24) who was a smoker (38 µg of Gly-HSA bound to 69 µg of IgG) and as compared with age matched non-smoker individual (serum no. 3) (43 µg of Gly-HSA bound to 62 µg of IgG) (Figure 4B). Langmuir plot was used to determine the apparent association constant. The constants estimated by Langmuir plot were 1.18 × 10^−6^ M and 3.32 × 10^−7^ M for Gly-HSA with IgGs from IV-S and IV subjects, respectively. Similarly, 1.76 × 10^−6^ M and 4.83 × 10^−7^ M constants were computed for Gly-HSA with IgGs from III-S and III group subjects, respectively. 

## 3. Discussion

Currently, the world population has more young adults than elderly individuals, and the aging of this population is growing at an increased rate [23]. The populations of most developed countries continue to become older, as in the United States alone, it is estimated that there will be 70 million people over the age of 65 by the year 2030, representing almost 25% of the population [24]. Hence, it is of vital importance for clinicians and scientists to have a better insight and understanding of the mechanisms associated with aging. 

In this study, HSA was glycated and the biochemical changes in glycated samples were analyzed. First, the formation of an early glycation product, ketoamine was estimated, and it was found that there were increased amounts of ketoamine in Gly-HSA as compared to the native form. Generation of in vitro carbonyl content in the glycation reaction of HSA was also confirmed, as intermediary products of glycation as well as oxidative stress markers. Furthermore, the final and advanced glycation product of glycation ‘pentosidine’ was also detected in the reaction sample. Generation of free radicals has a direct connection with the formation of AGEs and its precursors. These free radicals produced concomitantly with AGEs exert structural damage and functional losses of biomolecules (lipids, DNA, and proteins) [25]. 

The daily intake of AGEs inhibitors in natural products can play a beneficial role in preventing the pathogenesis of age-related diseases [22]. Therefore, in this study, garlic extract and EGCG a derivative of natural product (green tea) have been screened as potential inhibitors of AGE-pentosidine formation. Significant inhibitory anti-glycation activity was achieved by both garlic extract and EGCG, and validated previous findings, which showed that natural products and their derivatives can inhibit glycation [20,21,22]. It is believed that the daily intake of natural products that potentially are anti-oxidants and inhibit AGEs can, thus, play a beneficial role in preventing age-related pathogenesis. 

Glycation is a continuous process that contributes to the process of aging. Increased oxidative stress and formation of AGEs may also be associated with aging [13,26]. It has been previously observed that protein glycation products are deposited in tissues of living organisms with aging [27]. Various age-related deteriorative changes are due to protein degradation, deterioration of functional proteins, and development of toxic molecules leading to activation of inflammatory pathways. All of these changes often remain unknown to the individual unless clinical manifestation occur [22]. Analyses were performed for the presence of intermediary (carbonyl contents) and advanced (pentosidine) glycation products in the serum samples of the individuals without any signs and symptoms of any diseases among different age groups. From our results, it has been ascertained that elderly individuals with a history of smoking exhibited presence of significantly higher amounts of serum carbonyl content and pentosidine as compared to the age matched non-smoker subjects. Pentosidine levels were analyzed using two different methods; (a) pentosidine specific fluorescence and ELISA based detection of serum pentosidine concentration.

Both natural product (garlic extract) and a derivative of a natural product (EGCG) have been evaluated as inhibitors against the formation of AGEs. However, some of the synthetic compounds are toxic and exert considerable amounts of side effects; therefore, the use of natural products is more promising as potential AGE inhibitors [28]. Garlic extract and green tea derivate EGCG exhibited significant inhibitory effects in the formation of AGEs. 

Alterations in various clinical parameters are associated with aging, such as blood glucose, HbA1C, systolic and mean blood pressure, hypertension, and serum triglyceride concentrations [4,5,6,29]. According to the data, elderly individuals who were smokers exhibited a substantial increase in the levels of fasting blood glucose (FBG) and HbA1C as compared to age matched elderly who were non-smokers.

The aging process has a great influence on the immune system of an individual. A series of complex events usually leads to remodeling/restructuring that involves both the innate and the adaptive immune systems. These events are collectively termed as “immunosenescence”, or “immune dysregulation”. Immunosenescence include three main events: (a) a decreased immune response, (b) an increase in oxidative stress and inflammatory responses, and (c) generation and release of autoantibodies [30]. These previous findings are further supported by our immune assay results in which all the samples were screened for the presence of circulatory autoantibodies against Gly-HSA. Significant increase in Gly-HSA-Abs was detected in individuals who were 60–80 years old and were smokers (III-S), as well as elderly who were smokers (IV-S) when compared with age matched non-smoking individuals. This suggests that smoking is an important contributing factor that augments the production of circulatory autoantibodies with aging. It is well established that the immune system deteriorates with increase in age [31,32], and the detection of autoantibodies increases progressively with the aging process [26,27,29,30]. It has also been established that there are autoantibodies with diverse specificities (rheumatoid factor, antinuclear antibodies, and anti-cardiolipin antibodies) found in healthy elderly individuals over the age of 80 years. High titers of non-organ specific antibodies were found to be present in the elderly. Antibodies, such as rheumatoid factor, antinuclear antibodies, and anti-cardiolipin antibodies were detected in 14%, 31%, and 51% of healthy individuals over 80 years of age, respectively, in comparison to less than 2% in the non-elderly population [19]. 

Gly-HSA-Abs specificity was ascertained by Inhibition ELISA and results were consistent with the direct binding ELISA. Significantly high Gly-HSA-Abs were detected in elderly who were also smokers (IV-S) as compared to their age matched non-smoker subjects (IV). There was a remarkable difference (18.2%) in maximum percent inhibition in group IV-S subjects as compared to III-S subjects. No substantial difference (5.2%) of maximum percent inhibition was calculated in non-smoker subjects who were more than 80 years of age (group IV) and the subjects aged between 60 and 80 years (group III). This proves that smoking is an important contributing factor that is enhanced in the generation of circulatory Gly-HSA-Abs in elderly individuals. 

Antigen and antibody interactions were also calculated using Langmuir plot. Elderly individuals with smoking habits showed higher recognition of serum IgGs to Gly-HSA as compared with non-smoking individuals. The higher and specific recognition of Gly-HSA to serum IgGs might be due to the increased age (>80 years) and smoking, which concomitantly exaggerated protein oxidation that induced serum albumin structural alterations; thus, generating unique epitopes that might play role in the production of Gly-HSA-Abs. It is well documented that smoking enhances oxidative stress and hence formation of ROS [33,34]. Better recognition of Gly-HSA by serum IgG of elderly is indicative of the participation of glycated proteins in asymptomatic aging pathogenesis. These Gly-HSA-Abs can be considered as a potential marker for age-related disorders in elderly. 

## 4. Materials and Methods

### 4.1. Study Subjects

Sera were obtained from individuals without signs and symptoms of any disease, with their prior consents and investigations were carried out according to the Declaration of Helsinki (1964). Responsible ethical committee approved the experiments. 

In this study, the individuals were divided into groups. Each group was further sub divided based on smokers and non-smoker individuals. Each group comprised of twenty-five individuals (*n* = 25). Groups were distributed on the basis of age (years) of the subjects and smoking habits (Table 2). The distribution are as follows; group I (21–40 years); group I-S (smokers and 21–40 years); group II (41–60 years); group II-S (smokers and 41–60 years); group III (61–80 years); group III-S (smokers and 61–80 years); group IV (>80 years); and group IV-S (smokers and >80 years). Any subject showing any apparent signs or symptoms of disease were excluded from the study. Subjects from any past diseases or complications were also excluded from this study. Individuals who were more than 80 years of age were considered as elderly.

Criteria for smokers: Cigarette smoking history was assessed on the basis of a self-report questionnaire filled by subjects and completed before the experimental work was started on the samples. Smoking was quantified using number of cigarettes smoked per day. The following questions about smoking were used in the questionnaire: (1) Do you smoke? (2) If you smoke, when did you start to smoke regularly? (3) How much do you smoke (cigarette smoke on an average per day)? (4) If you do not smoke, have you previously smoked? After complete analysis of the questionnaires, individuals who smoked four or more than four cigarettes per day were considered as smokers in this study. The durations of smoking are given in Table 1. Individuals who smoked three cigarettes or less, and also non-regular smokers, were not included in the study. Non-smokers, who did not smoke throughout their life. 

Fasting blood sugar estimations and determination of HbA1C levels were assessed using well-known prescribed methods (glucose oxidase method and capillary electrophoresis method respectively) regularly used in the clinics. 

### 4.2. Glycation 

HSA was glycated as discussed before with slight modifications [25]. Briefly, 20 µM of HSA was dissolved in 20 mM phosphate buffered saline (PBS), pH 7.4, filtered through a 0.2 µm Millipore filter and incubated with 0.05 M d-glucose under sterile conditions in capped vials for 10 weeks at 37 °C in the dark. Solution of HSA without glucose served as control. After incubation, the solutions were extensively dialyzed against PBS and stored at –80 °C before use. Protein concentration was measured spectrophotometrically at 280 nm using absorption coefficient ε1% 280 nm = 5.3 M^−1^·cm^−1^ [35].

### 4.3. Detection of Ketoamines by NBT Reagent 

Glycation of HSA was detected using nitroblue tetrazolium (NBT) method [36]. Bovine serum albumin (BSA) (10 mg/mL) was incubated with 0.5 M glucose for 15 days at 37 °C in 20 mM PBS, which resulted in the complete modification of protein, with the subsequent formation of ketoamines [36]. N-HSA and Gly-HSA samples (50 µL) were added to the wells of microtiter plates in duplicate. One hundred microliters of NBT reagent (250 µM in 0.1 M carbonate buffer, pH 10.35) was added to each well and incubated at 37 °C for 2 h. The plate was read by ELISA reader at 550 nm. The amount (mol/mol of protein) of Gly-HSA in the sample was calculated using the standard curve constructed with glycated BSA.

### 4.4. Determination of Protein-Bound Carbonyl Contents

Protein-bound carbonyl contents were estimated in serum samples of individuals from all the groups according to Levine et al. [37]. Protein bound carbonyl content of in vitro modified non-glycated and glycated HSA samples were also estimated. The results were expressed as the number of nanomoles of carbonyl groups per milligram of sample protein, using the equation: ε379 nm = 22,000 M^−1^ cm^−1^ [35]. 

### 4.5. Assay of AGE-Fluorophores

Pentosidine a well-known AGE molecule was detected in in vitro glycated and non-glycated HSA samples. Pentosidine specific fluorescence was analyzed at an excitation wavelength of 375 nm and the fluorescence peaks were observed in the wavelength ranges from 330–420 nm [38]. The slit width for excitation and emission were 10 nm. For this assay, Gly-HSA and N-HSA samples were used at the concentration of 60 µM.

### 4.6. Serum Pentosidine Detection by ELISA

Pentosidine from serum samples of the individuals from all groups were detected using a competitive ELISA kit (FSK pentosidine ELISA kit; Fushimi Pharmaceutical, Kagawa, Japan) as described earlier [39]. In brief, serum samples were added to pronase and incubated at 55 °C for 1.5 h. After incubation, the mixture was heated in boiling water for 15 min to inactivate the enzyme. The samples were washed with PBS containing 0.5 mL/l Tween 20 buffer. After washing pentosidine specific antibodies and pretreated serum sample were added to each well and incubated at for 1 h at 37 °C. Rabbit anti-human IgG polyclonal antibodies labelled with peroxidase were then added and incubated for 1 h at room temperature. Color developing reagent containing 0.5 mg/mL of 3,3′5,5′-tetramethylbenzidine (TMB) was added to each wells. The reaction was stopped 10 min later by adding 100 µL of TMB. The absorbance was measured within 10 min at 450 and 630 nm (main and reference wavelength, respectively). The standard curve for pentosidine was obtained by measuring standard pentosidine solution and used to analyze the pentosidine concentration in serum samples [39]. 

### 4.7. Inhibition in AGE Formation by Natural Products

Natural product extract from garlic, and derivatives from green tea epigallocatechin-3-gallate (EGCG), were used as anti-glycating agents, and the amount of AGEs were estimated using the competitive ELISA kit [39]. Briefly, glycation reactions were co-cultured with various garlic extract (0–100 mg/mL) [40] and EGCG (0–100 µM) [41,42] concentrations throughout the reaction. All of the reaction samples were carried out under similar conditions and same period of time (10 weeks). Glycation reaction of HSA without any inhibitor considered as control.

### 4.8. Cytokines IL-1β and IL-6 Estimation

Serum levels of IL-1β and IL-6 were determined by quantitative sandwich immunoassay using commercially available kits (R&D System, Minneapolis, MN, USA) with a sensitive assay of < 0.5 pg/mL and 0.7 pg/mL, respectively. Samples were assayed in duplicate. 

### 4.9. Direct ELISA

Direct binding ELISA was performed on flat-bottomed 96-well polystyrene immunoplates (Maxisorp TM; Nalge Nunc International, Roskilde, Denmark), as described previously [43,44]. Briefly, microplates were coated with 100 µL of Gly-HSA or N-HSA (20 µg/mL) and were incubated for 2 h at room temperature and then at 4 °C overnight. ELISA plates were washed using TBS-T buffer (20 mM Tris, 2.68 mM KCl, 150 mM NaCl, pH 7.4) containing 0.05% Tween-20. Then, unbound sites were blocked with 150 µL/well of 2% skimmed milk in TBS (10 mM Tris, 150 mM NaCl, pH 7.4), and the microplates were incubated for 4–6 h at room temperature. After incubation, microplates were washed with the washing buffer and serum samples were diluted to 1:100 in TBS-T buffer. These diluted serum samples were added (100 µL/well) in each wells and incubated for 2 h at room temperature and then overnight at 4 °C. Unbounded serum antibodies were washed away and bound antibodies were assayed using anti-human IgG alkaline phosphatase conjugate using *p*-nitrophenyl phosphate as a substrate. The absorbance of each well was estimated at 410 nm on an automatic microplate reader. The cut-off value for ELISA estimation was established to be ≤ 0.05 absorbance recorded at 410 nm.

### 4.10. Competition ELISA

Antigenic specificity of the Gly-HSA was estimated by competition ELISA [43,44]. Increasing concentrations of Gly-HSA and N-HSA (0–20 µg/mL) were allowed to interact with a constant amount of serum autoantibodies for 2 h at room temperature and overnight at 4 °C. After incubation, the immune complex formed was incubated in the wells (instead of the serum taken in direct binding ELISA) and the bound antibody levels were detected as in direct binding ELISA. The percent inhibition was calculated using the formula:

Percent inhibition = [1 − (A_inhibited_/A_uninhibited_)] × 100 (1)

where, A_inhibited_ is the absorbance at 20 µg/mL of inhibitor concentration and A_uninhibited_ the absorbance at zero inhibitor concentration.

### 4.11. Quantitation of Affinity of Antibodies from Elderly Individuals

Formation and quantitation of immune complexes between the Gly-HSA and circulatory antibodies were evaluated [45]. Serum IgGs were isolated from serum number 5 of IV-S group and from serum number 24 from group III-S subjects. Serum IgGs were also isolated from non-smokers group IV (serum number 10) and III (serum number 3). The 100 µg of IgGs were incubated with different amount (0–80 µg) of antigens (Gly-HSA) in a total volume of 500 µL. The reaction mixture was incubated at 25 °C for 2 h and overnight at 4 °C. Immune complexes were pelleted, washed with PBS, and dissolved in 250 µL NaCl (1 N). Free and bound protein were estimated by colorimetric methods [35]. The data was used to calculate affinity of the antibodies from elderly using Langmuir plot [46].

### 4.12. Statistical Analysis

All results were expressed as the mean ± SD. Multiple comparisons between data were done using software OriginPro 8.5 followed by Student’s *t* test. The *p* value for significance was set at < 0.05. Correlation analyses were performed using Microsoft Office Excel 2010. Pearson correlation tests were adopted to analyze the correlations between parameters.

## 5. Conclusions

In vitro glycation of HSA leads to the formation of ketoamine, carbonyl compounds, and AGE (pentosidine). Significant inhibition of in vitro AGE formation was achieved using garlic extract and EGCG. Presence of serum carbonyl compounds and pentosidine suggests gluco-oxidation occurs in elderly. Significant sero-prevalence of Gly-HSA-Abs were detected in elderly individuals who were also smokers as compared to the non-smokers of same age groups. Strong correlations were found between Gly-HSA-Abs and FBG, serum levels of pentosidine, carbonyl content, and IL-6 in different age groups (II, II-S, III, III-S, IV, and IV-S). Hence, increase in age and smoking might exert gluco-oxidative stress and pro-inflammatory cytokines production that induce plasma protein structural alterations and, hence, gradual formation of cryptic epitopes. These new epitopes might induce generation of circulatory autoantibodies in the elderly. Hence, such asymptomatic elderly individuals are immunologically imbalanced. Use of natural products in everyday life might be helpful in minimizing age-related pathophysiological changes in elderly.

## Figures and Tables

**Table 1:** Chemical and structural characterizations of native human serum albumin (N-has) and glycation of human serum albumin (Gly-has).

Parameters	N-HSA	Gly-HSA
Ketoamine (mol/mol of HSA)	0.25 ± 0.1	6.4 ± 0.3 **
Carbonyl content (mol/mg of HSA)	0.1 ± 0.02	3.11 ± 0.4 ***
Pentosidine Fluorescence (AU) Exc. at 275 nm	5.2 ± 0.7	247.5 ± 8.7 ***
Serum Pentosidine (µg/mL) ELISA method	0.0092 ± 0.0004	0.0863 ± 0.012 **

N-HSA was considered as standard in all the estimations. Each value represents arithmetic mean ± SD of three independent assays. Ext. represents excitation emission wavelength. *t* test was adopted for the comparison between the two groups and significance is defined as ** *p*  <  0.01, *** *p*  <  0.001.

**Table 2:** Demographic and clinical characterizations of different individuals based on different age groups with or without smoking habits.

Groups (Age in Years) *n* = Subjects	Sex M/F	Fasting Blood Glucose (mg/dL)	HbA1_C_ (%)	Smoking Duration (years ± SD)	Serum Pentosidine (µg/mL ± SD)	Carbonyl Content (nmol/mg protein)
I (21–40) *n* = 25	16/09	83.0 ± 7.1	5.4 ± 0.3	―	0.0259 ± 0.0021	0.78 ± 0.15
I-S (21–40) *n* = 25	15/10	85.1 ± 7.1	5.5 ± 0.5	10.5 ± 5.1	0.0264 ± 0.0024	0.93 ± 0.14
II (41–60) *n* = 25	16/09	90.3 ± 8.5	5.6 ± 0.4	―	0.0262 ± 0.0030	0.84 ± 0.14
II-S (41–60) *n* = 25	16/09	94.2 ± 8.5	5.6 ± 0.3	16.9 ± 7.7	0.0269 ± 0.0029	1.2 ± 0.2
III (61–80) *n* = 25	15/10	94.8 ± 7.3	5.8 ± 0.5	―	0.0265 ± 0.0022	0.92 ± 0.22
III-S (61–80) *n* = 25	15/10	98.8 ± 8.3	5.9 ± 0.4	19.9 ± 9.7	0.0285 ± 0.0031 *	1.64 ± 0.4 **
IV (> 80) *n* = 25	25/25	106 ± 8.5	6.3 ± 0.7	―	0.0271 ± 0.0027	1.21 ± 0.3
IV-S (> 80) *n* = 25	25/25	118 ± 9.5	6.9 ± 0.8	29.9 ± 11.7	0.0321 ± 0.0029 *	2.42 ± 0.5 ***

For pentosidine and carbonyl content assay, each serum sample was run in duplicate. All data are given in mean ± standard deviation (SD). Signs ‘S’ represents smokers. *t* test was adopted for the comparison between the two groups and significance is defined as * *p < 0.05*, ** *p*  <  0.01, *** *p*  <  0.001, when compared between smokers and non-smokers with same age groups.

**Table 3:** Immunological characteristics of different individuals based on different age groups with or without smoking habits.

Groups	IL-6 (pg/mL)	IL-1β (pg/mL)	Maximum Percent Inhibition at 20 µg/mL	
			N-HSA-Ab	Gly-HSA-Ab
I	4.9 ± 0.66	1.1 ± 0.26	7.5 ± 1.3	7.8 ± 2.3
I-S	4.7 ± 0.56	0.99 ± 0.19	7.9 ± 2.6	9.4 ± 2.4
II	5.2 ± 0.67	1.16 ± 0.21	8.0 ± 2.2	12.4 ± 3.1
II-S	5.1 ± 0.61	1.21 ± 0.23	7.9 ± 2.2	19.4 ± 4.1
III	5.3 ± 0.65	1.18 ± 0.25	8.8 ± 1.8	21.1 ± 3.3
III-S	6.4 ± 0.69 *	1.28 ± 0.28	8.9 ± 1.8	33.1 ± 4.0 *
IV	5.2 ± 0.54	1.20 ± 0.19	9.3 ± 2.1	29.3 ± 4.3
IV-S	7.2 ± 0.65 **	1.62 ± 0.21 *	9.1 ± 2.1	51.3 ± 4.1 ***

For inhibition, ELISA and cytokine assay samples were run in duplicate. All data are given as mean ± standard deviation (SD). ‘N-HSA-Abs’ represents autoantibodies against N-HSA. Sign ‘S’ represents smokers. *t* test was adopted for the comparison between the two groups and significance is defined as * *p* < 0.05, ** *p*  <  0.01, *** *p*  <  0.001, when compared between smokers and non-smokers within same age groups.

**Table 4:** Correlation analysis between Gly-HSA-Ab levels and serum fasting blood glucose (FBG), pentosidine, carbonyl contents, and cytokine levels.

		I	I-S	II	II-S	III	III-S	IV	IV-S
		Correlation coefficient (r)							
**Gly-HSA-Abs**	FBG	−0.12	0.20	0.92 ***	0.99 ***	0.90 ***	0.98 ***	0.95 ***	0.98 ***
	Pentosidine	0.79 **	0.74 **	0.90 ***	0.99 ***	0.96 ***	0.99 ***	0.96 ***	0.99 ***
	Carbonyl content	0.72 **	0.72 **	0.93 ***	0.93 ***	0.98 ***	0.99 ***	0.97 ***	0.97 ***
	IL-6	0.56 *	0.48 *	0.92 ***	0.98 ***	0.96 ***	0.82 **	0.94 ***	0.90 ***

Correlation analyses were performed using Microsoft Office Excel 2010. Pearson correlation tests were adopted to analyze the correlations between parameters. Significance is defined as * *p < 0.05*, ** *p*  <  0.01, *** *p*  <  0.001.
